# Comparison of Demographic Life‐History Traits of the Snake‐Eyed Skink: Observations From an Island and a Mainland Population

**DOI:** 10.1002/ece3.70699

**Published:** 2025-01-15

**Authors:** Mehtap Ağdağ, Mehmet Zülfü Yıldız, Abdullah Altunışık

**Affiliations:** ^1^ Zoology Section, Department of Biology, Graduate Education Institute Adıyaman University Adıyaman Turkey; ^2^ Zoology Section, Department of Biology, Faculty of Arts and Sciences Adıyaman University Adıyaman Turkey; ^3^ Department of Biology, Faculty of Arts and Sciences Recep Tayyip Erdoğan University Rize Turkey

**Keywords:** body size, island, morphometry, sexual dimorphism, skeletochronology

## Abstract

The life‐history traits of animals are influenced by several factors. It has been proposed that key factors such as competition, predation pressure, and resource availability may differ between mainland and island populations of the same species. In this context, our study focused on an island (Yayla, Cyprus) and mainland (Hassa, Türkiye) populations of the snake‐eyed skink, *Ablepharus budaki*. This study aimed to reveal both intra‐population and inter‐population relationships in terms of mean age, longevity, age at maturity, body size, and sexual dimorphism. Our results show that lizards in the island population had longer lifespans and higher mean ages than the mainland population. Nevertheless, both populations were comparable in terms of mean body size. We also concluded that island individuals reach sexual maturity approximately 1 year later than their mainland conspecifics, and sexual dimorphism in terms of size is observed only in the mainland population. This study, offering initial demographic insights into the non‐mainland population of the species, provides the reason for additional research in this field.

## Graphical abstract

Our results show that lizards residing in the island population, where predation pressure and competition are reduced and food is more abundant, had longer lifespans and mean ages than the mainland population. Nevertheless, both populations were comparable in terms of mean body size. We also concluded that island individuals reach sexual maturity approximately 1 year later than their mainland conspecifics and sexual dimorphism in terms of size is observed only in the mainland population.

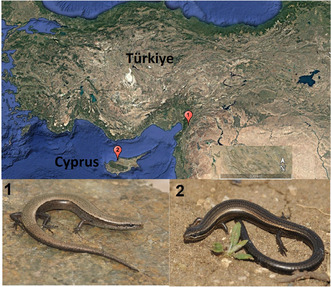

## 1 Introduction

Life aspects such as growth, development, and reproduction, which vary considerably between populations along environmental and altitudinal gradients, constitute the life history of animals. Any observed differences in life‐history traits between populations of the same species can be attributed to environmental factors. Even genetically determined aspects can be altered due to genetic expression (Adolph and Porter 1993; Mesquita, Costa, et al. 2016; Mesquita, Faria, et al. 2016). Palkovacs (2003) proposed a life‐history theory that three main aspects, such as competition, predation pressure, and resource availability, differ between island and mainland populations, describing the tendency of small animals to evolve and have larger body sizes on islands in comparison to their mainland conspecifics (island rule: Meiri 2007). Two alternative hypotheses have been proposed to explain the changes in the body size of animal species on islands, whether bigger or smaller than on the mainland. The first hypothesis suggests that reduced predation pressures lead to lower extrinsic mortality, causing larger body sizes (Case 1978). The second hypothesis points to altered resource availability as a major factor influencing these life‐history variations (Palkovacs 2003; Li et al. 2011). For example, it is expected that an island's fauna will exhibit greater access to food and therefore larger body size due to the reduction in predation pressure and competition between species that inhabit it (Case 1978; Uller et al. 2019). It has already been reported that environmental factors such as food availability, quality, and temperature differences between habitats can greatly increase stress and have a significant impact on the life history of organisms (Mesquita and Colli 2010; Reniers et al. 2015).

Lizards are ideal organisms for demographic life‐history studies because of the differences in lifestyle they exhibit and the ease with which their life‐history characteristics can be quantified (Ballinger 1979). In particular, it is relatively straightforward to measure age‐specific survivorship probabilities and reproductive characteristics for a multitude of lizard species in their natural habitats, using phalanxes without harming them (Comas et al. 2020; Ma et al. 2022; Altunışık et al. 2024). Some of the differentiations that occur in the tissues of living organisms during their lifetime are recorded in the histomorphological structure of bone in reptiles, providing information about lifespan, age at maturity, and physiology (Kidov, Ivanov, et al. 2023; Tatlı and Altunışık 2024). Skeletochronology is a commonly employed method for analyzing the age distribution of numerous cold‐blooded species. It relies on the observation of indicators called “growth markers” or “Lines of Arrested Growth” (LAGs), which develop in different types of bones, including phalanges, femurs, tibias, and humerus. The emergence of these markers is a result of decreased metabolic activity within bone tissue during estivation or hibernation (Gibbons and McCarty 1983). Using the information obtained from the bones (skeletochronology), we can quantify and compare the life‐history characteristics of lizard populations from different regions and climates (Altunışık et al. 2023; Kidov, Ivanov, et al. 2023; Göğebakan, Mermer, and Çiçek 2024).

Demographic life‐history traits of skins, the family Scincidae, are limited (Kidov, Kondratova, et al. 2023; Altunışık et al. 2024). *Lamprolepis smaragdina* , Emerald skink from Negros Island, Philippines (Alcana 1966), *Plestiodon obsoletus*, Great plains skink from Kansas, USA (Hall and Fitch 1971), *Heremites vittatus*, Bridled Mabuya from Sivas, Türkiye (Kalayci et al. 2018), and *Ablepharus kitaibelii*, Juniper Skink from Bulgaria (Vergilov, Tzankov, et al. 2018; Vergilov, Necheva, et al. 2018) are some of the species on which age studies have been conducted. Moreover, no data are available on the life‐history parameters of the snake‐eyed skink, *Ablepharus budaki*, Göçmen, Kumlutas, and Tosunoglu 1996, a member of this family, outside the mainland. The distribution area of the snake‐eyed skink is restricted to Türkiye, Cyprus, Syria, and Lebanon (Lymberakis et al. 2009). We examined whether the age structure, age at maturity, longevity, body size, and sexual dimorphism of the snake‐eyed skink differed in two separate populations, one on the mainland and one on an island. We also aimed to compare these characteristics between sexes within and between populations.

## 2 Materials and Methods

### 2.1 Studied Species and Sites

The Yayla locality is located 5 km west of Güzelyurt district, in the northwest of the Turkish Republic of Northern Cyprus. The region mainly consists of agricultural lands, but in the coastal areas, it has natural vegetation in dune habitats. According to the macroclimate classification of Cyprus, it falls under the “semi‐arid” climate zone. As a Mediterranean island, Cyprus experiences hot and dry summers, while winters are mild with low rainfall. The average annual temperature of Cyprus is 19.0°C. The area, dominated by a Mediterranean climate, is characterized by shrubs and annual herbaceous plants. Although the specimens were mostly collected from natural areas near the coast, they were also gathered from natural areas remaining between fields in an area of approximately 10 km^2^. Hassa district is located in the north of Hatay province, 80 km from Hatay, on the eastern slopes of the Amanos Mountains. The district shares a 24 km border with Syria. The Mediterranean climate prevails in Hassa, with temperatures ranging from 4°C to 33°C throughout the year. The average annual rainfall is 763 kg/m^2^. The area is dominated by forest and maquis vegetation. The specimens were collected actively from between the leaves or from under stones within an area of approximately 40 km^2^.

**FIGURE 1:**
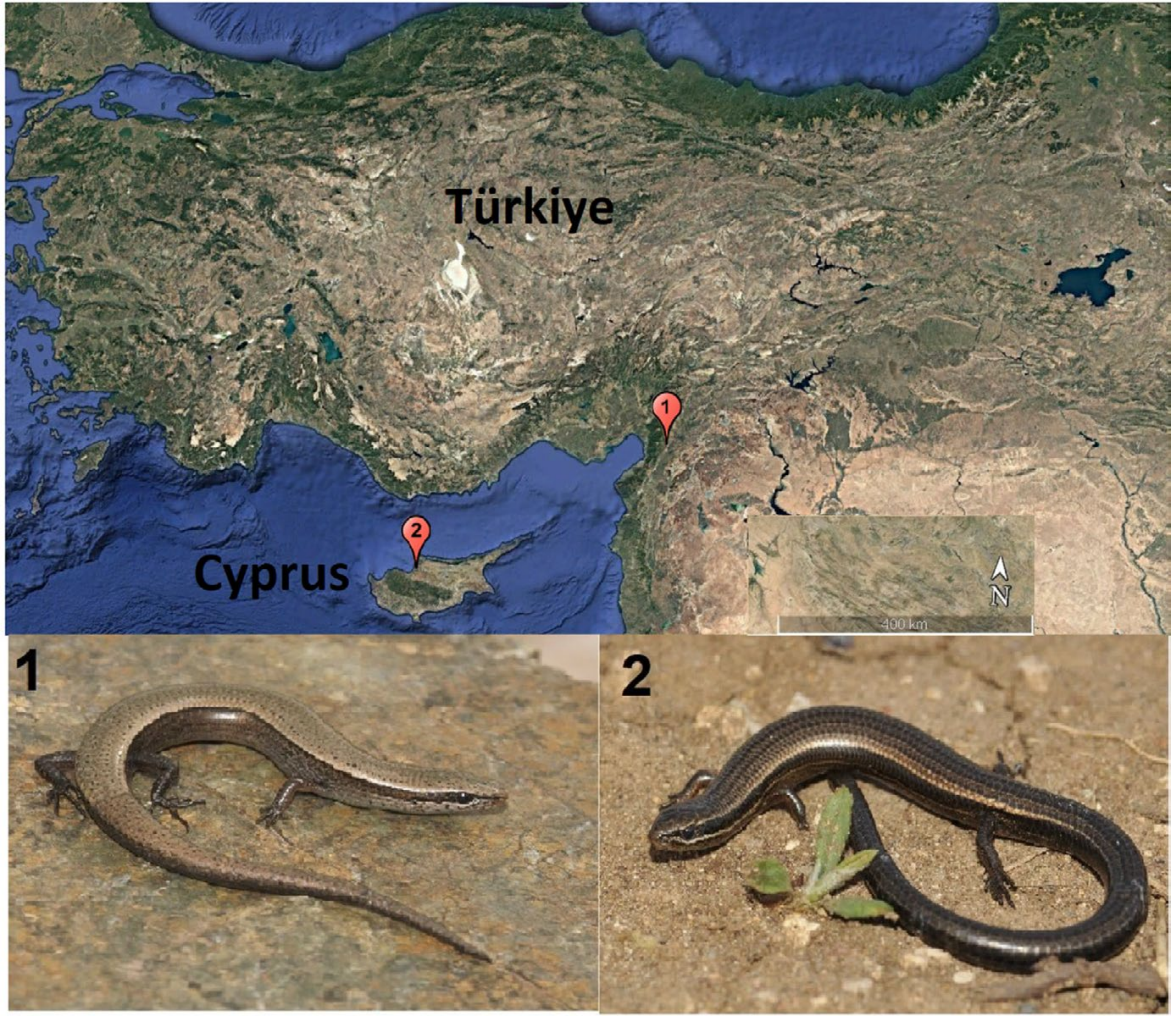
Sampling locations of the snake‐eyed skink (*Ablepharus budaki*) in this study. 1: Hassa, Hatay, Türkiye; 2: Yayla, Cyprus (Google Maps).

Specimens collected between 1994 and 2019 and conserved in the Adıyaman University Zoology Museum were used for this study. No new specimens were collected from nature to avoid harming skink populations for this study. A total of 59 specimens (mainland: 12 males, 11 females, and six juveniles from Hassa, Türkiye; island: 13 males, 14 females, and three juveniles from Yayla, Cyprus) were used for this study (Figure 1). Sampling was performed with the permission of the Adıyaman University Faculty of Medicine Animal Ethics Committee (Date: 29.04.2011, decision number: 2011/071). The sex of the museum specimens was determined by examining the gonads under a stereo microscope. The testes in males are light‐colored and smooth; in females, the ovary is yellowish, and the eggs are more or less visible (Sömer 2022). The coloration of specimens observed during fieldwork exhibited a wide range, from light orange to dark red. The majority of cases occurred in males and exhibited greater intensity during the breeding period. It has been observed that the coloration is limited to the ventral surface of the body, excluding the ventral regions of the head and tail. Alternatively, it may occur in the gular region (comprising the body and tail), or it may be restricted to the ventral surface of the tail. In juveniles, in contrast, the red coloration gradually darkens on both the upper and lower surfaces of the tail as it extends from the nostril to the tip of the tail (Göçmen, Kumlutas, and Tosunoglu 1996; Sömer 2022). Specimens' SVL (snout‐vent length; distance from the tip of snout to cloaca) was measured using a digital caliper sensitive to 0.01 mm (Altunışık 2023). Subsequently, following Smirina's guidelines (Smirina 1994), the fourth toe of the right hind limb was excised with a pair of scissors and preserved in a 70% ethanol solution until histological analysis.

### 2.2 Age Determination

The distance between two neighboring LAGs is a good predictor of individual development in a particular year. The age at which sexual maturity was reached was determined by looking for a distinct reduction in the interval between two successive LAGs (Ryser 1998).

The skeletochronological investigation was carried out using the modified techniques of Altunışık et al. (2021), following the methods of Castanet (1994). The second phalanx, preserved in 70% ethanol, was first soaked in distilled water for a day and then subjected to a decalcification process for approximately 2 h using a 5% HNO_3_. After obtaining cross‐sections with an 18 μm thickness using a Shandon Cryostat microtome, Ehrlich's hematoxylin dye was applied to them and left for 10 min.

**FIGURE 2:**
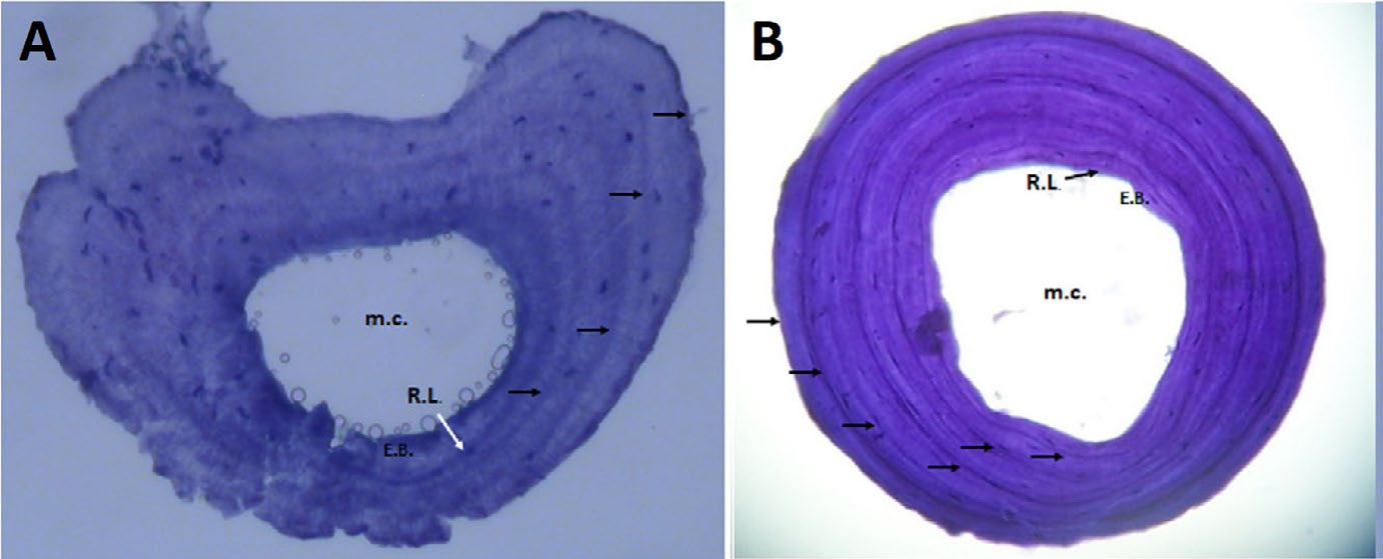
Cross‐sections (18 μm thick) at the diaphysis level of the phalange bone of the snake‐eyed skink specimens from the mainland (A) and island (B). E.B., endosteal bone; m.c., marrov cavity; R.L., resorption line.

Sections exhibiting rather narrow bone marrow cavities and the widest periosteal bone were specifically chosen to minimize the error that may arise from endosteal resorption and then immersed in a glycerine solution (Castanet and Smirina 1990). Using a light microscope equipped with a Pixera digital camera at 10× and 20× magnifications, the selected samples were observed and photographed (Figure 2). All authors conducted an independent count and confirmation of the LAGs subsequent to reviewing all captured images (Altunışık 2018; Altunışık, Üçeş, and Yıldız 2022).

### 2.3 Statistical Analyses

The experimental data (SVL, Age) underwent statistical analysis utilizing SPSS version 22.00. The data's normality was assessed via a Shapiro–Wilk test. For comparing sexes or populations, the non‐parametric Mann–Whitney *U* test was employed. Spearman correlation coefficient was used to investigate the association between SVL and age. The mean values were provided with corresponding standard error (SE) or standard deviations (SD). Sexual size differences were estimated using the Sexual Dimorphism Index (SDI) formulated by Lovich and Gibbons (1992).

$$\mathrm{SDI}=\left(\frac{\text{size}\;\left(\mathrm{SVL}\right)\;\text{of larger}\;\mathrm{sex}}{\text{size}\;\left(\mathrm{SVL}\right)\;\text{of smaller}\;\mathrm{sex}}\right)-1$$

The von Bertalanffy growth model was utilized to ascertain growth trends, as is the case in other research (Guarino 2010; Altunışık et al. 2023). The von Bertalanffy growth has the following generalized formula:

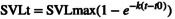




In this formula “SVLt” refers to size at age *t*, “SVLmax” indicates the asymptotic highest SVL, *e* is the Euler's number (2.718…), “*k*,” the growth coefficient, defines the curve's shape, and hatching age (*t*_0_) is the age at metamorphosis. Since the hatching size of *A. budaki* is not available we used the hatching SVL (19.78 mm) of *A. kitaibelii* (Glavaš et al. 2018).

## 3 Results

### 3.1 Demographic Parameters and Body Size in the Mainland Population (Hassa, Türkiye)

In the mainland population, the age of adult specimens varied from 3 to 6 years in both male (average: 4.00 ± 1.04) and female specimens (average: 4.54 ± 1.12) (Table 1). There was no statistical difference in the average age between males and females within this group (Mann–Whitney *U* test; *p* = 0.26). Endosteal resorption, resulting in partial erosion of the periosteal bone at the edge of the medullary cavity, was observed in cross sections of 70.37% of the adult specimens.

**TABLE 1:** Descriptive statistics of the island and the mainland population of *Ablepharus budaki*.

Traits	Island population		Mainland population		*p*
	Mean ± SD	Range	Mean ± SD	Range	
**Adults**					
*Male*	*N*:13		*N*:12		
SVL	39.07 ± 3.92	33.02–44.75	35.27 ± 3.96	29.59–42.29	**0.025** *
Age	5 ± 0.81	4–6	4 ± 1.04	3–6	**0.033** *
*Female*	*N*:14		*N*:11		
SVL	39.80 ± 2.85	36.14–45.44	40.08 ± 3.98	34.81–48.36	0.839
Age	5.71 ± 0.82	4–7	4.54 ± 1.12	3–6	**0.013** *
**Juvenile (unknown sex)**	*N*:3		*N*:6		
SVL	22.55 ± 1.91	20.41–26.38	27.85 ± 0.53	25.91–29.54	
Age	1	1	1.50 ± 0.22	1–2	
**Total**	*N*:27		*N*:23		
SVL	39.45 ± 3.36	33.02–45.44	37.57 ± 4.59	29.59–48.36	0.103
Age	5.37 ± 0.88	4–7	4.26 ± 1.09	3–6	**0.001** *

*Note:* *Bold *p*‐values less than 0.05 indicate a significant difference between the mainland and island populations.

**FIGURE 3:**
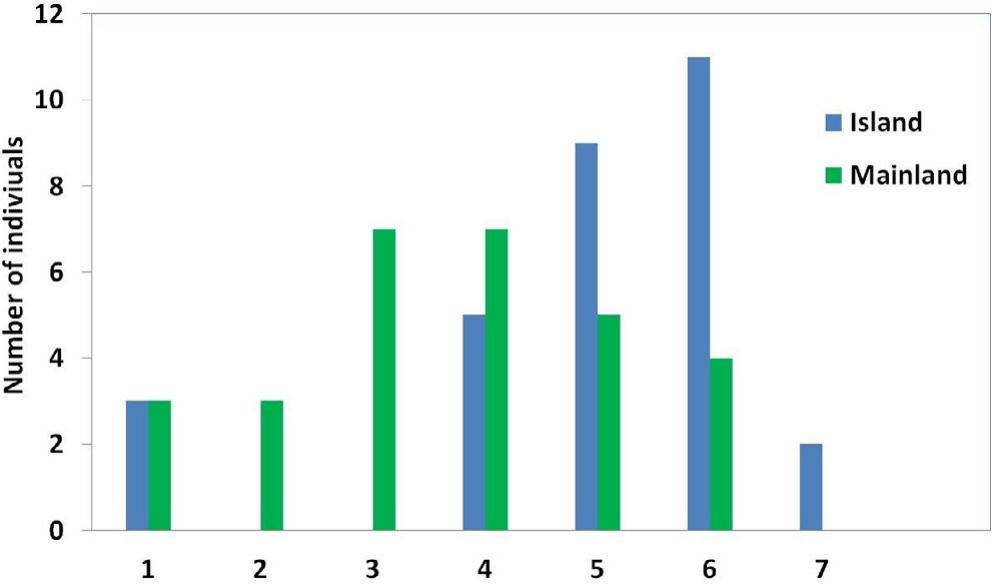
Age distribution in the island and mainland populations of the snake‐eyed skink.

The SVL of juveniles of the mainland population ranged from 25.91 to 29.54 mm (mean: 27.85 ± 0.53 mm). The six juveniles in the population were aged one or two (Table 1). On the mainland, most individuals were either 3 or 4 years old, comprising 48.27% (*n* = 14; Figure 3). Sexual maturity was reached at 2–4 (mean: 2.73 ± 0.67) years for breeding individuals. The SVL of male individuals of the mainland population ranged from 29.59 to 42.29 mm (mean: 35.27 ± 3.96 mm) and from 34.81 to 48.36 (mean: 40.08 ± 3.98) in females. In the mainland population, females were found to have statistically greater body length than males (Mann–Whitney *U* test; *p* < 0.01). This finding was corroborated by the SDI calculated as 0.13. In this population, a positive correlation (Spearman correlation coefficient; r = 0.745, *p* < 0.05) was observed between SVL and age.

### 3.2 Demographic Parameters and Body Size in the Island Population (Yayla, Cyprus)

The island population consisted of adult specimens aged 4–6 years in males (mean: 5.00 ± 0.81) and 4–7 years in females (mean: 5.71 ± 0.82) (Table 1). The average age of females was greater than males (Mann–Whitney *U* test; *p* < 0.05). The age of the three juveniles in this population was 1 year (Table 1). On the island population, most of the individuals were 6 years old, comprising 36.66% of the island population (*n* = 11; Figure 3). In the island population, it was determined that individuals reach maturity between 3 and 5 years of age. In general, the average age at sexual maturity of this population was determined as 3.37 ± 0.91 years. Endosteal resorption was observed in cross sections of 65.22% of the adult specimens of this population.

The SVL of male individuals of the island population ranged from 33.02 to 44.75 mm and from 36.14 to 45.44 mm in females. There was no meaningful disparity in SVL between females (mean: 39.80 ± 2.85 mm) and males (mean: 39.07 ± 3.92 mm) (Mann–Whitney *U* test; *p* = 0.65), supported by a week SDI of 0.01. A positive correlation was observed between SVL and age values of the island population (Pearson correlation coefficient; r = 0.696, *p* < 0.05).

### 3.3 Comparison of the Mainland (Hassa) and Island (Yayla) Populations

The mean age of both males and females of the island population was higher than that of their mainland counterparts (males and females; Mann–Whitney *U* test; *p* < 0.05). In addition, when all individuals are evaluated together, regardless of sex, the average age (5.37 years) of the island population was found to be statistically higher than the average age (4.26 years) of the mainland population (Mann–Whitney *U* test; *p* < 0.01).

The SVL of male individuals of the island population ranged from 33.02 to 44.75 mm and from 36.14 to 45.44 mm in females. The SVL of male individuals of the mainland population ranged from 29.59 to 42.29 mm and from 34.81 to 48.36 mm in females. The mean SVL of male individuals of the island population was higher than that of male individuals of the mainland population (Mann–Whitney *U* test; *p* < 0.05). However, the mean SVL value of female individuals was not significantly different between the two populations (Mann–Whitney *U* test; *p* = 0.979). On the other hand, there was no statistically significant difference between the two populations in terms of mean SVL, regardless of sex (Mann–Whitney *U* test; *p* = 0.06). On a species basis (regardless of population), females (39.93 ± 3.32 mm) were found to be larger in mean SVL than males (37.25 ± 4.32 mm) (Mann–Whitney *U* test; *p* < 0.05).

**FIGURE 4:**
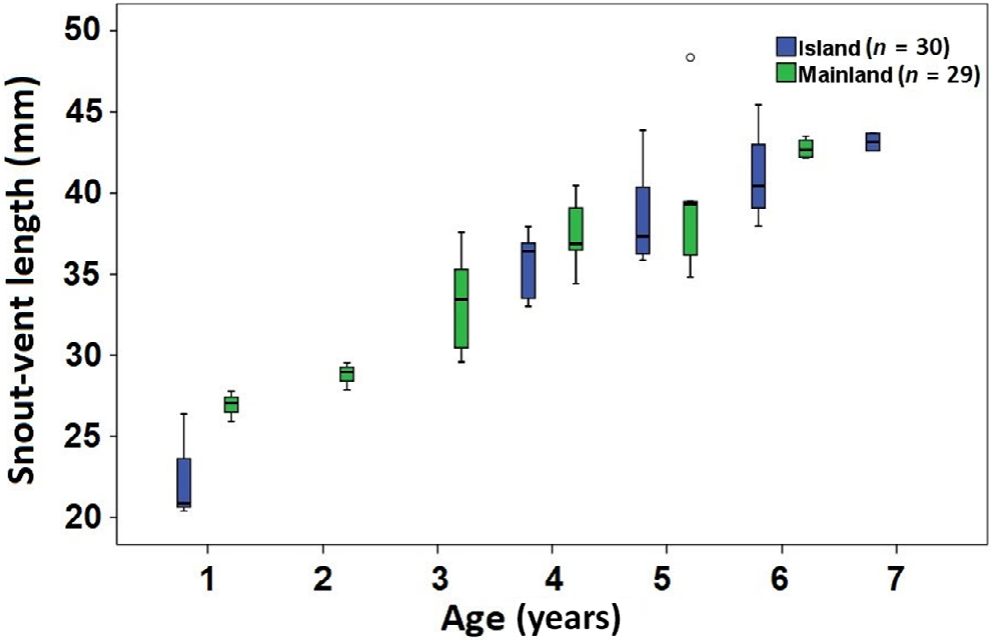
Age–SVL relationship in island and mainland populations of the snake‐eyed skink. Error bars represent the standard deviation.

**FIGURE 5:**
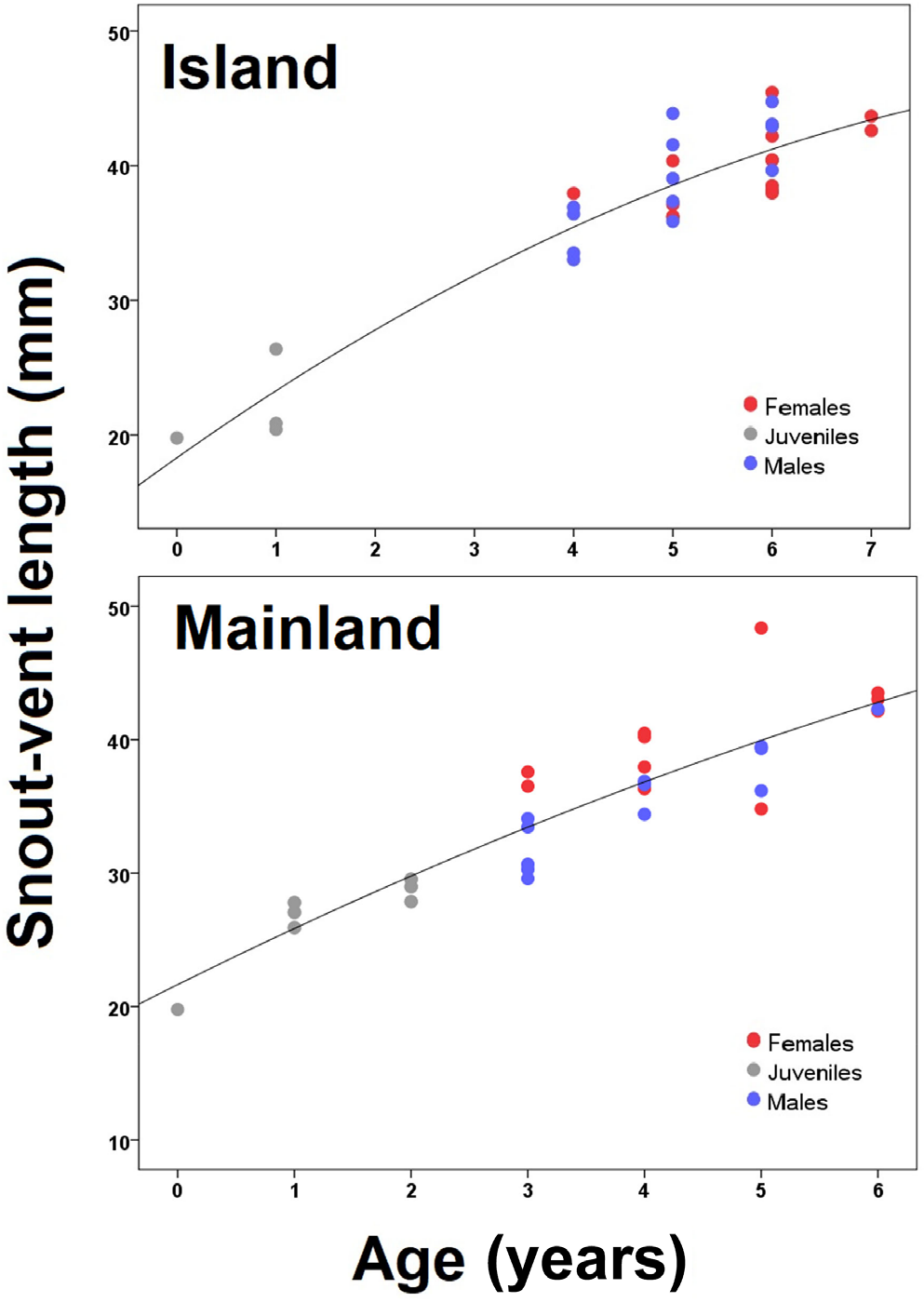
Relationship between SVL and age in island and mainland populations of the snake‐eyed skink with the von Bertalanffy growth model.

A positive correlation was observed between SVL and age values similarly in both groups (Figure 4). The von Bertalanffy growth model showed a fit that accurately represented the relationship between SVL and age in island (SVLasymptotic = 49.20 mm, *k* = 0.31) and mainland (SVLasymptotic = 53.00 mm, *k* = 0.19) populations (Figure 5).

## 4 Discussion

This research was conducted with the central aim of comparing the demographic parameters of *A. budaki* populations across Cyprus (island) and Türkiye (mainland). Extensive demographic research within the genus *Ablepharus*, including this skink species, has been limited, as indicated in previous literature (Kalayci et al. 2018 (*n* = 18); Mermer et al. 2020 (*n* = 95); Vergilov, Tzankov, and Zlatkov 2018 (*n* = 48); Wapstra and Swain 2001) (Table 2). The average age of *A. budaki* examined in this study, regardless of sex and site, was calculated as 4.86 years with a small sample size (*n* = 11). The age of adult individuals in the island population ranged from 4 to 7 (mean: 5.37 ± 0.88) and from 3 to 6 (mean: 4.26 ± 1.09) in the mainland population. Findings from the interpopulational comparison of the age structure among the populations investigated in this study indicated that the average age of *A. budaki* in the island population surpassed that of the mainland population.

**TABLE 2:** Body size (SVL) and mean age in some representative Scincidae family species.

Species	*N*	SVL (mm ± SD)		Mean age ± SD		Region	References
		Male	Female	Male	Female		
*Lamprolepis smaragdina*		56.5		1–5		Negros Island, Philippines	Alcala (1966)
*Plestiodon obsoletus*	29	70–130		6.2		Kansas, USA	Hall and Fitch (1971)
*Carinascincus ocellatus*		62.65–82.59	64.57–88.94	1–12		Tasmania, Australia	Wapstra and Swain (2001)
*Chalcides chalcides*	40	89.76 ± 22.75	143.50 ± 23.25	13.82 ± 9.96 (months)	27.22 ± 9.61 (months)	Southern Italy	Guarino (2010)
*Eulamprus leuraensis*		52–84	66–86	2.25	3.00	Southeastern Australia	Dubey et al. (2013)
*Ablepharus budaki*	11	34.24	4.83	Türkiye	Yıldırım et al. (2017)		
*Ablepharus chernovi*	7	41.23	5.85				
*Heremites vittatus*	18		71.25 ± 7.20		3.53 ± 0.87	Sivas/Türkiye	Kalayci et al. (2018)
*Ablepharus kitaibelii*	48	41.37	45.79	2.20	2.71	Bulgaria	Vergilov, Tzankov, and Zlatkov (2018)
*Chalcides ocellatus*	95	79.20 ± 13.40	82.10 ± 14.22	6 ± 1.61	5.80 ± 2.76	South Türkiye	Mermer et al. (2020)
*Ablepharus bivittatus*	59	30.5–49.7	30.6–54.4	2.6 ± 1.24	3.6 ± 1.15	Ardabil, Iran	Kidov, Kondratova, et al. (2023)
*Eumeces schneiderii*	41	125.50	131.08	8.31	8.52	Mediterranean populations, Türkiye	Altunışık et al. (2024)
*Eumeces schneiderii*	36	107.10	118.31	6.85	8.13	Continental populations, Türkiye	
*Ablepharus budaki*	50	37.24 ± 4.31	39.92 ± 3.32	4.52 ± 1.04	5.20 ± 1.11	Türkiye‐Northern Cyprus	This study

Lifespan and the timing of sexual maturity are fundamental life‐history traits in any organism (Altunışık, 2017). These factors are intimately related to its survival and reproductive capabilities, playing a key role in determining the organism's fitness (Cabezas‐Cartes, Boretto, and Ibarguengoytia 2018). When the age at sexual maturity of the mainland and island populations was analyzed, it was found that the former population reached sexual maturity earlier. Many studies confirm that individuals who reach sexual maturity later have a longer lifespan (Tinkle, Wilbur, and Tilley 1970; Castanet and Baez 1991; Roff 1992; Andreone and Guarino 2003). In this study, individuals belonging to the island population were found to have longer lifespans and mean ages, supporting this trend.

According to the general average SVL results of the populations; although the SVL of females is statistically larger than males, there is no significant difference between the average values of males (mean = 39.07 ± 3.92 mm) and females (39.80 ± 2.85) individuals in the island population. However, Göçmen, Kumlutas, and Tosunoglu (1996) also reported the mean SVL of males as 39.74 mm and the mean SVL of females as 48.00 mm in the subspecies identification for *A. budaki*. This supports the difference in SVL between the sexes in our study. Differences in body length between sexes within a species represent a foundational trait crucial for understanding essential biological characteristics (Denoël et al. 2009). Competitive selection mechanisms propel the sizes of male and female individuals in divergent directions, driven by factors such as competition for resources, habitat utilization, reproduction, and sexual selection (Blanckenhorn 2005). Size‐dependent sexual dimorphism (SSD) manifests as a developmental process occurring prior to puberty, elucidating the intricate interplay between sexual maturity, fertility, developmental stages, and lifespan (Barot et al. 2004; Day and Taylor 1997). Within this study, the sexual dimorphism favoring females pertains specifically to fertility, a trait intricately linked to sexual selection, as corroborated by numerous prior studies (Vitt and Cooper Jr 1986; Shine 1989; Hews 1990; Vincent and Herrel 2007; Altunışık 2023). In this study, the body size of the males of *A. budaki* from the island population was higher than that of the congeners from the mainland population. In females, the average body size of both populations was similar. Regardless of sex, island and mainland populations are similar (39 vs. 37 mm) in mean length. It has been reported that lizards do not show a consistent pattern of size evolution on the mainland and islands or show a pattern opposite to the island rule (Meiri 2007). Meiri (2007) found that small lizards on islands became smaller than their mainland counterparts, while large lizards (especially carnivorous lizards) became even larger, contrary to the predictions of the island rule. He also found no trends consistent with the island rule when maximum snout‐vent length was used. From this perspective, the size similarity of the lizards in the two *A. budaki* populations seem to be consistent with the literature.

Studies that delve into the life‐history traits of ectothermic organisms often center on investigating the interplay between age and body size. While specific species—such as *Chalcides chalcides* (*n* = 40), Three‐toed skink (Guarino 2010), *Chalcides ocellatus* (*n* = 95), Ocellated Skink (Mermer et al. 2020), and *Mediodactylus heterocercus* (*n* = 138), Asia Minor Thin‐toed Gecko (Altunışık et al. 2022)—exhibit a robust and statistically significant positive correlation between these factors, other species such as *Macroscincus cocte*, Cabo Verde Giant Skink (Andreone and Guarino 2003), *Eulamprus leuraensis*, Blue Mountain Water Skink (Dubey et al. 2013), and *Phoenicolacerta laevis*, Lebanon Lizard (Bülbül et al. 2021) have been documented without a significant relationship. Echoing this overarching pattern, *A. budaki* demonstrates substantial body size augmentation with age across both the island and the mainland populations.

## 5 Conclusions

Providing comparative data on longevity, age structure, age at sexual maturity, body size, and sexual dimorphism in an island and a mainland population of snake‐eyed skinks will facilitate an enhanced ecological understanding of this lizard species. Consequently, lizards residing in the island population had longer lifespans and higher mean ages than in the mainland population. Nevertheless, both populations were comparable in terms of mean body size. We also concluded that island individuals reach sexual maturity approximately 1 year later than their mainland conspecifics, and sexual dimorphism in size is observed only in the mainland population. This study, offering initial demographic insights into the non‐mainland population of the species, provides the reason for additional research in this field.

## Author Contributions

**Mehtap Ağdağ:** conceptualization (equal), formal analysis (equal), investigation (equal), methodology (equal), software (equal). **Mehmet Zülfü Yıldız:** conceptualization (equal), data curation (equal), funding acquisition (equal), investigation (equal), project administration (equal), resources (equal), supervision (equal). **Abdullah Altunışık:** formal analysis (equal), investigation (equal), methodology (equal), supervision (equal), visualization (equal), writing – original draft (equal), writing – review and editing (equal).

## Conflicts of Interest

The authors declare no conflicts of interest.

## Data Availability

All data generated or analyzed during this study are included in this manuscript.
